# Strain-Induced Surface Interface Dual Polarization Constructs PML-Cu/Bi_12_O_17_Br_2_ High-Density Active Sites for CO_2_ Photoreduction

**DOI:** 10.1007/s40820-023-01309-w

**Published:** 2024-01-16

**Authors:** Yi Zhang, Fangyu Guo, Jun Di, Keke Wang, Molly Meng-Jung Li, Jiayu Dai, Yuanbin She, Jiexiang Xia, Huaming Li

**Affiliations:** 1 School of Chemistry and Chemical Engineering, Institute for Energy Research, School of the Environment and Safety Engineering, Jiangsu University, Zhenjiang, 212013 People’s Republic of China; 2 State Key Laboratory Breeding Base of Green Chemistry-Synthesis Technology, College of Chemical Engineering, Zhejiang University of Technology, Hangzhou, 310014 People’s Republic of China; 3 College of Science, and Hunan Key Laboratory of Extreme Matter and Applications, National University of Defense Technology, Changsha, 410073 People’s Republic of China; 4 Department of Applied Physics, The Hong Kong Polytechnic University, Hung Hom, Hong Kong People’s Republic of China; 5 School of Chemistry and Chemical Engineering, National Special Superfine Powder Engineering Research Center, Nanjing University of Science and Technology, Nanjing, 210094 People’s Republic of China; 6 Key Laboratory of the Ministry of Education for Advanced Catalysis Materials, Zhejiang Normal University, Jinhua, 321004 People’s Republic of China

**Keywords:** Bi_12_O_17_Br_2_, Porphyrin, CO_2_ photoreduction, Polarization, Active sites

## Abstract

The insufficient active sites and slow interfacial charge transfer of photocatalysts restrict the efficiency of CO_2_ photoreduction. The synchronized modulation of the above key issues is demanding and challenging. Herein, strain-induced strategy is developed to construct the Bi–O-bonded interface in Cu porphyrin-based monoatomic layer (PML-Cu) and Bi_12_O_17_Br_2_ (BOB), which triggers the surface interface dual polarization of PML-Cu/BOB (PBOB). In this multi-step polarization, the built-in electric field formed between the interfaces induces the electron transfer from conduction band (CB) of BOB to CB of PML-Cu and suppresses its reverse migration. Moreover, the surface polarization of PML-Cu further promotes the electron converge in Cu atoms. The introduction of PML-Cu endows a high density of dispersed Cu active sites on the surface of PBOB, significantly promoting the adsorption and activation of CO_2_ and CO desorption. The conversion rate of CO_2_ photoreduction to CO for PBOB can reach 584.3 μmol g^−1^, which is 7.83 times higher than BOB and 20.01 times than PML-Cu. This work offers valuable insights into multi-step polarization regulation and active site design for catalysts.

**Supplementary Information:**

The online version contains supplementary material available at 10.1007/s40820-023-01309-w.

## Highlights

- Strain induces coupling in Bi_12_O_17_Br_2_ and Cu porphyrin-based monoatomic layer (PML-Cu), constructing Bi–O bonding interface in PML-Cu/BOB (PBOB).
- Surface interface dual polarization boosts internal electric field, promoting electron transfer.
- PML-Cu provides high density of dispersed active Cu sites in PBOB, enhancing CO_2_ photoreduction.

**Supplementary Information:**

The online version contains supplementary material available at 10.1007/s40820-023-01309-w.

## Graphical abstract

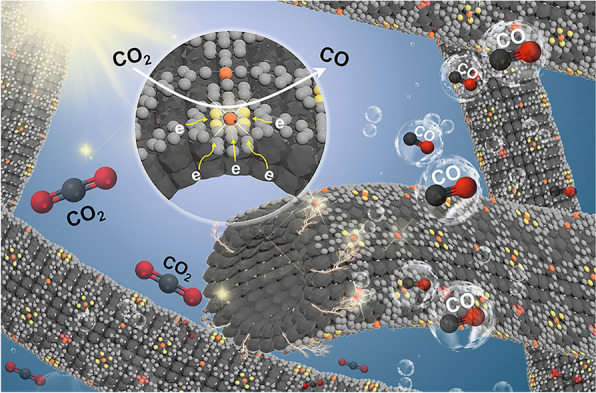

## Introduction

The massive consumption of fossil fuels has caused energy shortage, while the large emissions of carbon dioxide (CO_2_) has also posed serious atmospheric pollution, making an effective response to the energy and environmental crisis a major challenge for the world today [1–3]. Based on bionomics simulation from green plant, artificial photosynthesis provides a new response to the above problems [4, 5]. However, the high dissociation energy of CO_2_ (750 kJ mol^−1^) and the slow electron-proton coupling process in reaction make it difficult to fulfill the CO_2_ photoreduction efficiency for practical applications [6].

The process of photocatalytic CO_2_ reduction primarily involves three steps: the photoexcitation of the photocatalyst, the migration of photogenerated charges to the surface, and the subsequent reaction with CO_2_ adsorbed on the surface [7]. Today, a myriad of photocatalysts have been developed, including TiO_2_ [8], C_3_N_4_ [9, 10], BiVO_4_ [11, 12], CdSe [13–15] and others [16–18]. Among these, bismuth oxyhalide stands out as a promising material due to its crystal tunability and the high dependence of photocatalytic properties on its compositional structure [19, 20]. However, insufficient active sites and low charge separation efficiency limit their further application [21]. Although the construction of heterostructures enhances charge separation and introduces new exogenous active sites, the state and number of active centers are difficult to precisely regulate and the charge transfer process is still influenced by the contact interface [22]. For the targeted design of active sites in photocatalytic materials, porphyrin-based monoatomic layer (PML) materials show great prospects for application. PML can be viewed as a monoatomic lamellar layer formed by cross-coordination of the ends of the metalloporphyrin molecule with metal atoms. This two-dimensional ultrathin structure of the single-atom layer can strengthen the anisotropy of electron transfer, and the homogeneous periodic structure can make all metal catalytic sites highly uniform [23]. The chemical state of the central active site can be modulated by group and metal type, while the highly dispersed central metal maximizes atom utilization [24]. The introduction of PML onto the catalyst surface would be expected to enable the construction of the catalyst with a high density of active sites. Further, numerous research examples have demonstrated that the construction of a localized polarization field between materials can break their charge symmetry and promote efficient spatial separation of photogenerated electrons-hole pairs [25, 26]. Huang et al. optimized the electron migration process by coupling *V*_Ni_/*V*_Se_ double vacancies to form a spontaneous polarization electric field in NiSeS/ZnSe heterojunctions [27]. Liu et al. found that the construction of surface Bi–O vacancy pairs promoted the surface interface polarization of Bi_24_O_31_Br_10_ nanosheets with black phosphorus, and the interfacial electron bridge acted as a fast transfer path to enhance electron directional transfer [28]. Notably, the construction of structures similar to the above usually depends on the presence of surface defects, and the polarization regions are usually random. Strain engineering can cause surface bending deformation of the materials through tensile strain, which in turn leads to an increase in surface atomic escape energy [29]. The generation of a large number of surface vacancies causes widespread surface lattice mismatch, which results in the formation of unsaturated sites on the outer surface and offers the possibility of further loading of the materials and overall directed polarization.

Herein, strain-induced strategies were developed to achieve dual surface interface polarization. Specifically, Cu porphyrin-based atomic layers (PML-Cu) were constructed with Cu-TCPP as the substrate, and the assembly of PML-Cu with Bi_12_O_17_Br_2_ (BOB) was achieved by strain engineering, which triggers the interface-to-surface multistep polarization process of of PML-Cu/Bi_12_O_17_Br_2_ (PBOB). The strong coupling interface in PBOB motivates the rapid influx of photogenerated electrons from PBOB toward PML-Cu. The encapsulated PML-Cu over BOB provides the high density of active Cu sites and induces surface electron transfer toward them. As a result, the photocatalytic CO_2_ reduction performance of PBOB was significantly enhanced.

## Experimental Section

### Materials

Polyvinylpyrrolidone (PVP, AR) was purchased from Sigma-Aldrich Trading Co., Ltd. Bismuth nitrate pentahydrate (Bi(NO_3_)_3_·5H_2_O, AR), sodium bromide (NaBr, AR), mannitol (C_6_H_14_O_6_, AR), sodium hydroxide (NaOH, AR), anhydrous ethanol (ETOH, AR), formic acid (HCOOH, AR) and copper nitrate (Cu(NO_3_)_2_·3H_2_O, AR) were supplied from Sinopharm Chemical Reagent Co., Ltd. (China).

### Preparation of PML-Cu, BOB and PBOB Materials

#### Preparations of PML-Cu

PML-Cu: The material was synthesized according to previously report [23, 24]. *N*,*N*-dimethylformamide and ETOH were configured into a dispersion with a ratio of 3:1. Then, 2 mg Cu(NO_3_)_2_-3H_2_O, 40 μL formic acid and 10 mg PVP were added into 12 mL of the dispersion, record as solution A after mix. Five milligrams of Cu-TCPP (preparation method in Supporting Information) was added to 4 mL of the dispersion and mixed thoroughly as solution B. Subsequently, solution B was added to solution A with continuous stirring and the volume was fixed to 20 mL. The solution was transferred to a reaction vessel and heated at 80 ℃ for 2 h. After the reaction, the precipitate was washed several times and centrifuged. The precipitate was dispersed and transferred to a round bottom flask and dried by rotary evaporator. The resulting product was recorded as PML-Cu.

#### Preparations of BOB

Bi_12_O_17_Br_2_ (BOB) nanotubes: 0.243 g Bi(NO_3_)_3_-5H_2_O, 0.273 g mannitol and 0.18 g PVP were added to 15 mL deionized water and ultrasonically dispersed. 3 mL of KBr solution (0.17 M) was injected under stirring. Subsequently, the pH of the above solution was adjusted to 13 by 2 M NaOH solution. After stirring for 0.5 h, the solution was heated at 160 ℃ for 24 h. The product was centrifuged and washed several times with hot ethanol and water, dried by vacuum drying oven and noted BOB.

#### Preparations of PBOB

PML-Cu/Bi_12_O_17_Br_2_ (PBOB): 60 mg BOB was dispersed in a 150 mL round bottom flask containing 60 mL ethanol, and PML-Cu with a mass fraction of 5‰ was added to form a mixture. Then, it was stirred vigorously in an oil bath and heated at 85 ℃ for 12 h. The product was washed several times with deionized water and anhydrous ethanol. The precipitate was collected by centrifugation and dried in a vacuum drying oven, and recorded as PBOB.

## Results and Discussion

### Design Principle and Structural Characterizations

**Fig. 1:**
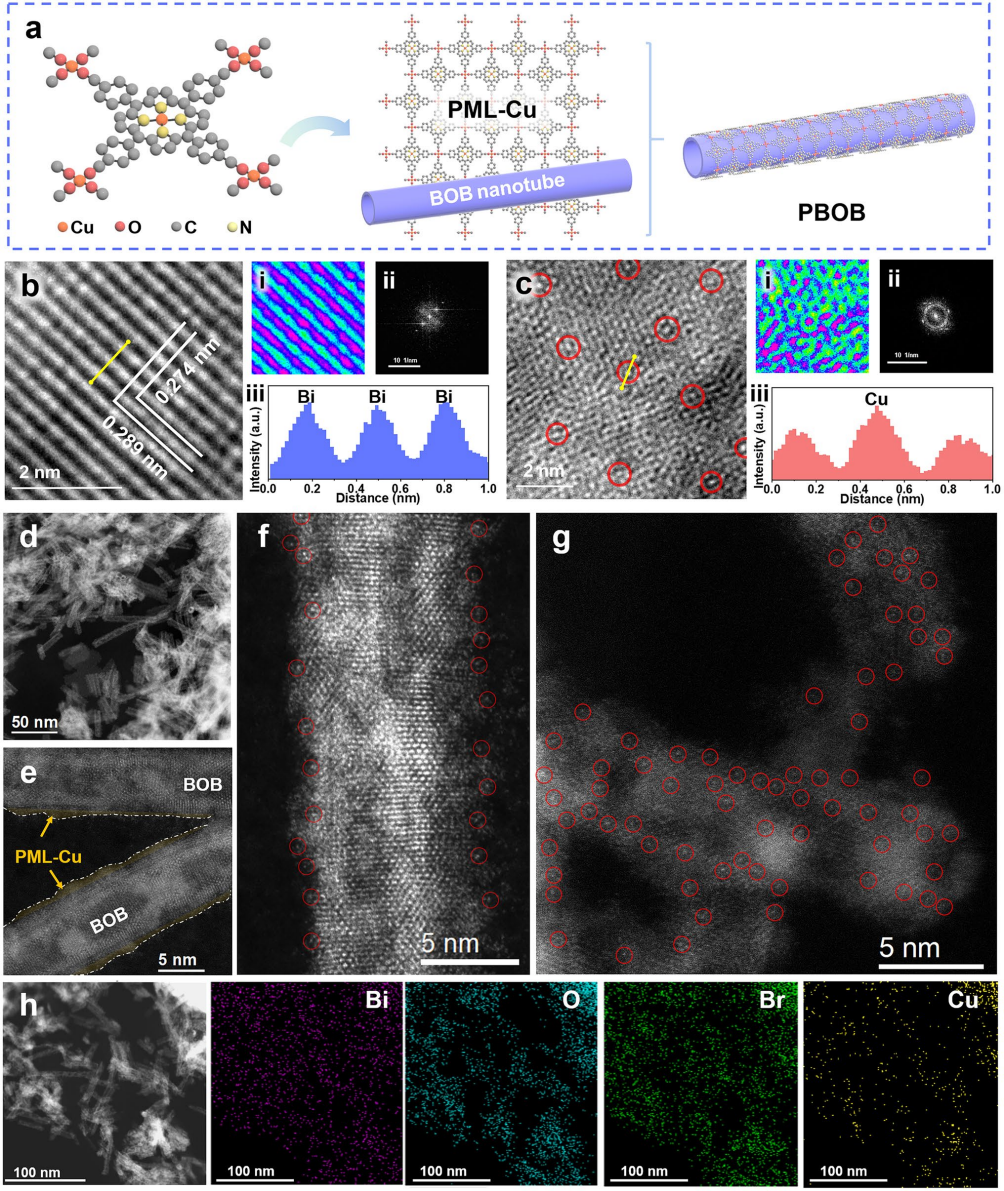
**a** Illustration for the formation of PBOB. HR-TEM of **b** BOB and **c** PBOB (**i** is the regional pseudo-color map, **ii** is the fast Fourier transformed pattern of lattice, **iii** is the intensity profile corresponding to the yellow arrow). **d–g** Atomic resolution HAADF-STEM images and **h** STEM-EDS mapping images of PBOB

The synthesis method of PBOB with surface high-density active sites is shown in Fig. 1a. The Cu porphyrin-based monoatomic layer (PML-Cu) was constructed using Cu-TCPP as a primitive. In this atomic layer structure, CuO_4_ and CuN_4_ act as metal junctions and ligand sites within the porphyrin ring, respectively, thus expanding and growning by bottom-up cross-linking reactions. Meanwhile, the ultrathin Bi_12_O_17_Br_2_ (BOB) nanotube structure was prepared by strain engineering. The surface distortion caused by its curved tubular structure allowed the escape of surface oxygen atoms and the formation of a large number of oxygen defects. Based on this, a large number of dangling bonds and surface coordination unsaturated atoms brought by the defective structure were utilized to realize the effective encapsulation of PML-Cu on the BOB surface, and the PML-Cu/Bi_12_O_17_Br_2_ (PBOB) was finally achieved. The compositional structure of the catalyst was explored by X-ray diffraction (XRD). As shown in Fig. S1, peaks of the materials at 29.0°, 32.7°, 45.0°, and 56.1° correspond to the (1 1 7), (2 0 0), (2 2 0), and (3 1 7) crystal planes, respectively, which match with the standard card of Bi_12_O_17_Br_2_ (JCPDS NO. 37-0701) [29]. The diffraction peak shapes of the PBOB are in accordance with that of BOB, indicating the capping of PML-Cu did not change the crystalline shape of the material. The effective combination of PBOB with PML-Cu was verified through Fourier transform infrared (FTIR) spectrum (Fig. S2). The vibrational peaks at 1406 cm^−1^ are correlated with δ(C–CN) of the porphyrin ring, and the peaks at 1003, 841 and 771 cm^−1^ corresponds to the vibrations of C_α_ − C_m_, ν(C_β_ − N), and Πp of the porphyrin backbone, respectively [30, 31]. The characteristic PML-Cu peaks at 1348 and 1003 cm^−1^ are similarly observed in PBOB, demonstrating the effective composite state form of the material.

The morphology of the catalysts was observed through high resolution transmission electron microscope (HRTEM). As shown in Figs. S3 and S4, PML-Cu exhibits an almost transparent flexible layered structure, while BOB shows a separate ultrafine tubular morphology and the diameter of the tubes is below 10 nm. The surface lattice of BOB was further measured (Fig. 1b and b(i)), which exhibits mutually perpendicular atomic arrangements. The narrower atomic spacing of 0.274 nm corresponds to the (2 0 0) or (0 2 0) crystal faces of Bi_12_O_17_Br_2_ crystals, whereas the wider atomic spacing stretches to 0.289 nm, correlating with an anisotropic lattice tensile strain parallel to the tube wall [32]. In Fig. 1c and c(i), the PBOB surface is in a disordered structural state, and the diffraction structure of material also changes from spots to rings (Fig. 1b(ii) and c(ii)), confirming the encapsulation of BOB by PML-Cu. The successive atomic structures of the BOB and PBOB surfaces were measured to obtain information about their intensities (Fig. 1b(iii) and c(iii)). The BOB surface is an arrangement of Bi atoms with the same liner, while the atomic liner of Cu in PBOB is higher than that of its neighboring coordinating C, N and O atoms and thus exhibits the higher atomic scale.

Aberration-corrected high-angle annular dark-field scanning transmission electron microscopy (HAADF-STEM) was applied to further resolve the state of Cu sites on the PBOB surface. From the HAADF-STEM images (Fig. 1d–f), PML-Cu with sub-nanometer thickness is encapsulated on the surface of BOB nanotubes. Many bright spots outside the BOB ordered lattice are observed at the edges of the PBOB nanotubes (voltage 80 kV), corresponding to Cu atoms in PML-Cu (marked by red circles) [33]. The high density of scattered Cu atoms with the distance of about 2 nm can be clearly observed on the surface of PBOB nanotubes (Fig. 1g), which is consistent with the TEM results. In Fig. 1h, energy-dispersive X-ray spectroscopy (EDS) elemental mapping analysis exhibits the uniform distribution of Bi, O, Br and Cu elements in PBOB. The above results indicate the effective establishment of high-density dispersed Cu atomic sites on the BOB surface.

### Surface Interface Dual Polarization Function

**Fig. 2:**
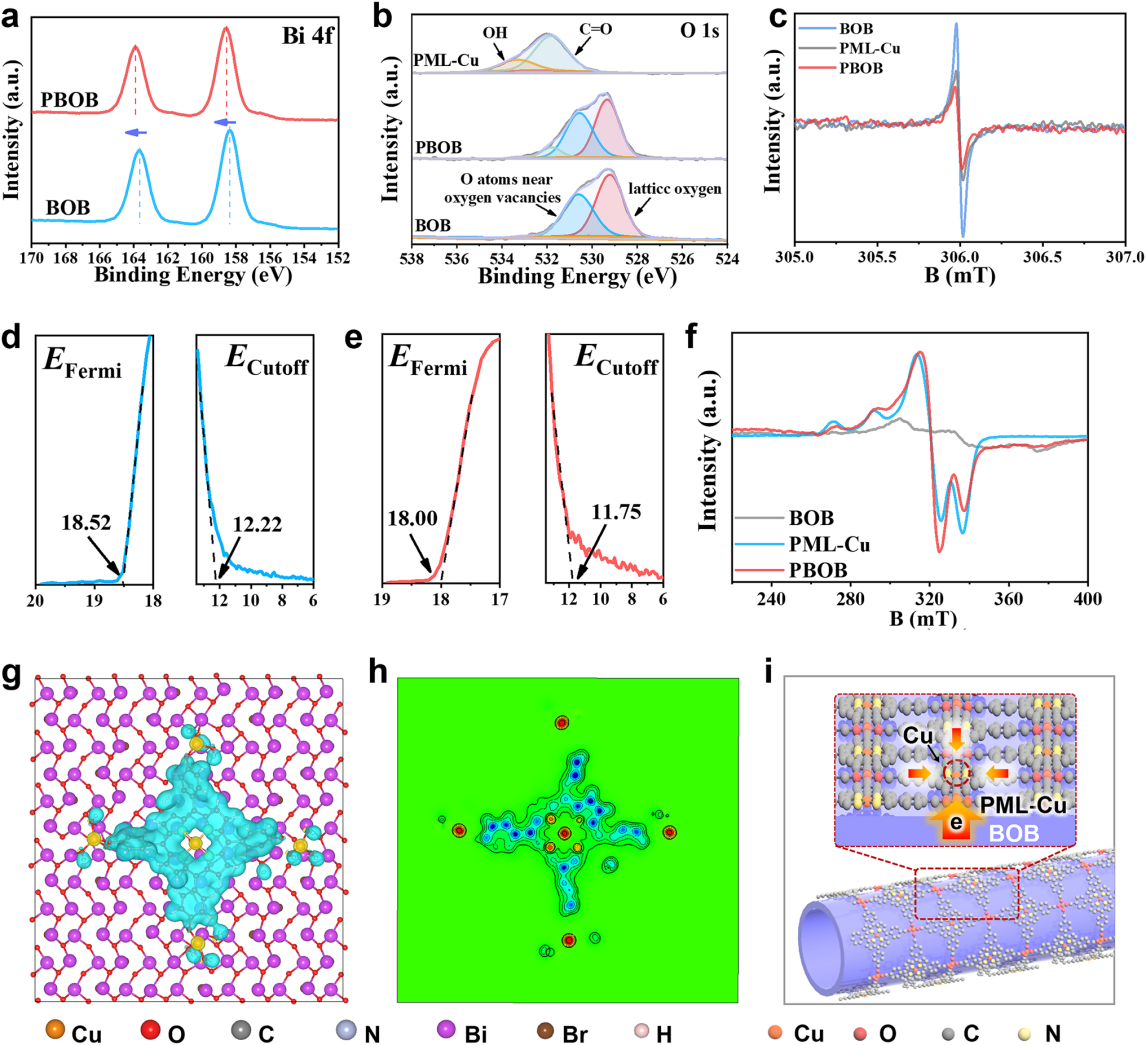
**a** Bi 4*f*, **b** O 1*s* XPS and **c** EPR spectra of the materials, UPS spectra of **d** BOB and **e** PML-Cu, **f** EPR spectra of the materials, **g** DFT calculation: Charge difference density image of PBOB and **h** ELF, **i** Schematic of surface interface dual polarization in PBOB

The structural variation of the surface interface of the material was further investigated. Raman analysis revealed the structural strain changes in materials. From Fig. S5, the Raman peak located at 141.3 cm^−1^ (Bi–O vibrational mode) is associated with magneto-electric coupling (A_1g_-3 mode) [34]. Upon constructing the PBOB, the peaks splitt toward high and low wave numbers, respectively, indicating the strong interaction between PML-Cu and the [Bi_12_O_17_] layer of the BOB. The surface structure of the material was explored through X-ray photoelectron spectroscopy (XPS) and electron paramagnetic resonance (EPR). As shown in Fig. 2a, b, Bi signature peaks located at 158.4 and 163.6 eV are shifted toward the high binding energy direction to 158.6 and 163.9 eV after the formation of PBOB [35]. Peaks attributed to lattice oxygen shifts from 529.2 to 529.4 eV [36], and the oxygen signal peaks associated with oxygen vacancies in BOB and the end-group oxygen signals in PML-Cu both shifts toward lower binding energies. Moreover, the oxygen vacancy concentration of PBOB in the EPR results (Fig. 2c) is significantly reduced compared to BOB and PML-Cu, revealing the effective occupation of BOB surface vacancies by PML-Cu [37]. However, such changes do not appear in the XPS spectra of Br (Fig. S6), suggesting that PML-Cu undergoes interfacial remodeling with the [Bi_12_O_17_] layer of BOB without affecting the halogen layer. In Fig. S7, the strong peak at 935.0 eV and the broad signal peak at 942.5 eV are the characteristic peak of Cu^2+^ and its companion peaks, respectively [38]. Although the Cu^2+^ signal is reduced due to the low introduction of PML-Cu in PBOB, the shift of the Rusche peak toward the low binding energy can still be observed. This indicats the interfacial reconstruction of BOB with PML-Cu can effectively regulate the chemical state of the surface metal sites and make the Cu sites to gain more electrons [39]. The work function (Φ) of the material was calculated by ultraviolet photoelectron spectroscopy (UPS) so as to study the interfacial charge migration. Here the energy of UV photon (*hν*) is 21.22 eV. Based on the formula: Φ = *hν* + *E*_Cut off_ − *E*_F_, the Φ of BOB is calculated as 14.92 eV, which is lower than that of PML-Cu (14.97 eV). The electrons in BOB are more likely to flow to PML-Cu, forming the internal electric field (IEF) in PBOB [40]. This IEF formed between the interface of BOB and PML-Cu can promote the interfacial polarization in PBOB and enhance the electron transfer between the inner layer and the surface layer. In the electrochemical impedance spectroscopy (EIS) (Fig. S8), the smaller Nyquist circle radius of PBOB reflects the enhancement of the charge mobility of the material and the electron transfer between interfaces becomes more efficient [41].

The surface electronic structure of the material was then explored. EPR tests were utilized to reveal information about the coordination of Cu atoms on the surface of the materials. With the exception of BOB, both PML-Cu and PBOB show significant Cu^2+^ signals. The PBOB signal intensity is higher than PML-Cu, indicating that it can accommodate more spin electrons. In addition, the weakening of the hyperfine splitting of PBOB verifies the enhanced dipole interactions of individual Cu porphyrin units on the surface of PBOB [42, 43]. The electron distribution on the PBOB surface was analyzed from the atomic level by theoretical calculations. PML-Cu was placed on a carrier consisting of multiple crystalline BOB crystals, then the charge difference density distribution of PML-Cu can be obtained. In Fig. 2g, h, the electrons are enriched in the Cu atomic sites (yellow portions) and form depletion regions in the porphyrin backbone (blue portions). The localized enhancement of the electron density of the Cu atomic sites in PBOB demonstrates that the surface polarization occurs over PBOB and induces electron transfer on PML-Cu to the active site Cu atoms. Based on the above result, the construction of PBOBs through strain-induced strategy promotes the surface interface dual polarization and achieves the modulation of surface Cu active sites (Fig. 2i). Specifically, this is a multi-step polarization process that the interfacial polarization between BOB and PML transfers electrons to the surface of PBOB. Meanwhile, the second-stage polarization of the surface PML urges electrons transfers to the Cu sites.

### Charge Migration and Energy Band Structure

**Fig. 3:**
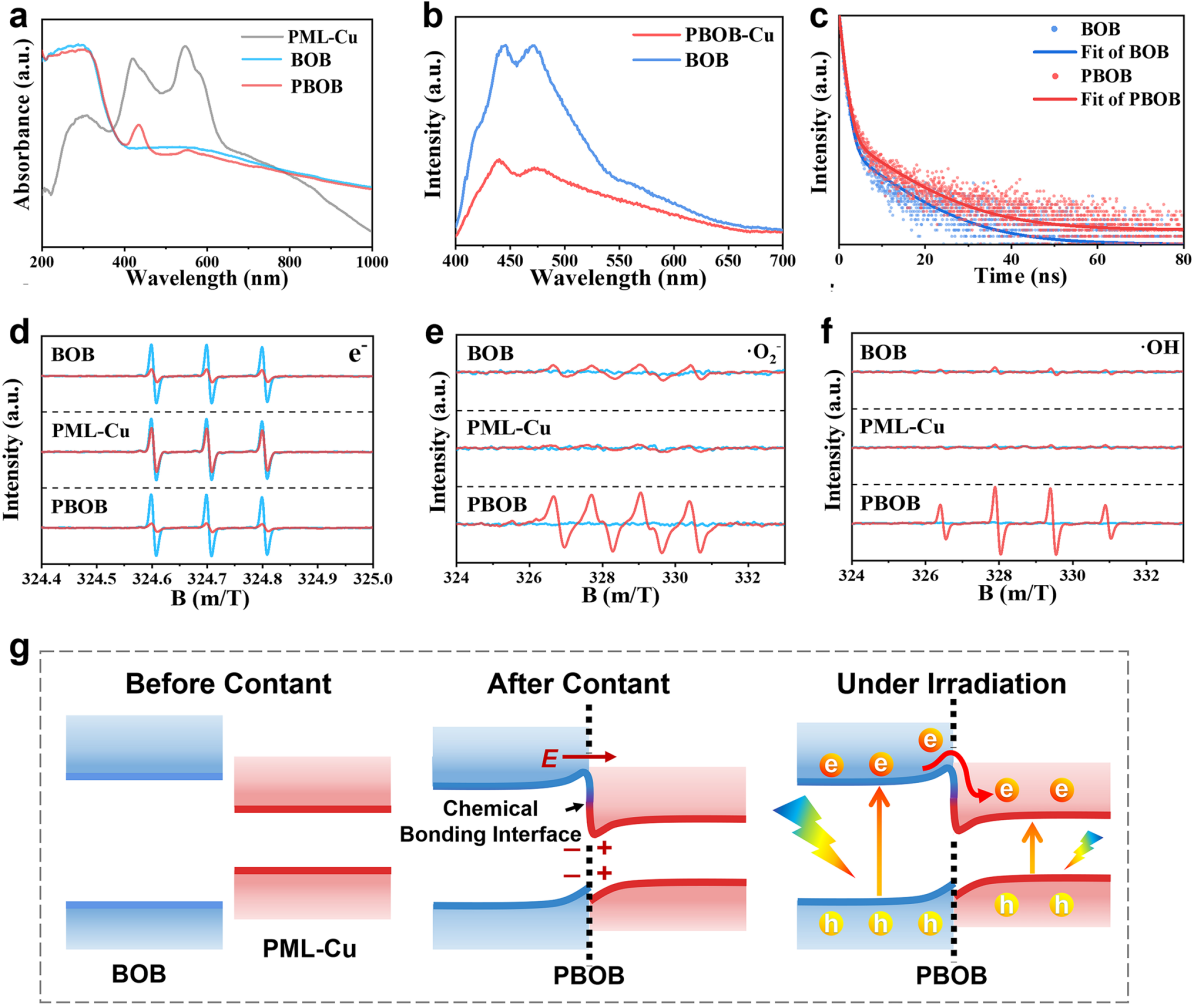
**a** UV–Vis diffuse reflection spectra, **b** PL spectra, and **c** time-resolved PL decay curves of the materials; ESR spectra for **d** e^−^, **e** ·O_2_^−^ and **f** ·OH of the materials; **g** Inferred electronic structure model of PBOB

The light absorption ability of the material has an important effect on its photogenerated electronic excitation. From the UV–Vis diffuse reflectance spectra of the materials (Fig. 3a), PML-Cu exhibits soret absorption at 420 nm and an α-band at 540 nm, indicating a highly out-of-domain state of the π-electrons in PML-Cu [44]. The absorption edge of BOB is located at 400 nm, and its structural defects induce the relevant in-band changes, causing a pronounced tail absorption in the range of 400–800 nm. After the constitution of PBOB, the in-band effect owing to defects decreases and the soret peak shifts to 435 nm, indicating that the interfacial remodeling of BOB with PML-Cu in PBOB further leads to the charge redistribution of porphyrin moieties. After conversion by Kubelka–Munk function (Fig. S9), the intrinsic band gaps of BOB and PBOB are 2.23 and 2.12 eV, respectively. In the XPS valence band spectra (Fig. S10), the VB of BOB is 1.21 eV and that of PBOB is 1.18 eV. Then, the values of the conduction bands of the material as well as the energy band structure diagrams are obtained in Fig. S11, demonstrating that the construction of the surface interface dual polarization in PBOB can still satisfy the CO_2_/CO reduction potential. Transient photocurrent test (Fig. S12) shows the photoresponsiveness of PBOB is highly improved.

Subsequently, the migration behavior of photogenerated electrons in the materials was investigated. From photoluminescence (PL) spectra of Fig. 3b, the strong fluorescence signal located at 420–540 nm is mainly generated by BOB. The significant quenching of the PBOB fluorescence signal indicates that the excited state electrons of BOB are effectively extracted [45]. This implies that the electrons can be rapidly transferred from BOB to PML-Cu under the dual surface interface polarization. Carrier lifetime curves in the time-resolved PL decay spectrum were fitted by a three-exponential function model. In Fig. 3c and Table S1, τ1, τ2, and τ3 represent the nonradiative process of transition of excited state electrons to lower energy states, the radiative process associated with electron–hole recombination, and the energy transfer process, respectively [35]. The decrease in the ratio of τ1 in PBOB verifies that the BOB photogenerated electrons in the polarized interface can be extracted rapidly before the energy state become lower. The increase in the ratio of τ2 and τ3 indicates that the interface polarization realizes an effective spatial transfer of charge in PBOB, which allows the photogenerated electrons to maintain the relaxation. In addition, the average carrier lifetimes of BOB and PBOB are 1.3426 and 1.5423 ns, and the longer lifetime of PBOB will promote efficient catalytic reactions.

The effect of surface interface dual polarization on the electron–hole separation ability of materials was analyzed through ESR tests (Figs. 3d and S13). Among them, TEMPO bursts both the more electron and hole signals of PBOB, demonstrating its enhanced surface effective charge concentration. Free radical test results similarly validate this observation. In Fig. 3e, f, the six DMPO-O_2_^−^ and four DMPO-·OH signals of PBOB are significantly enhanced, showing the stronger free radical generating capacity of PBOB. Notably, the energy band structure of PBOB satisfies the reduction potential for ·O_2_^−^ (− 0.33 eV) but not the oxidation production path for ·OH (2.29 eV). In this case, ·OH originates from the further conversion of ·O_2_^−^ (2·O_2_^−^ + 2H^+^  → 2·OH + O_2_), indicating the improvement in overall reduction capacity of PBOB [46]. This, the schematic diagram of possible electronic structures is given in Fig. 3g. The chemical bonding interface in PBOB realizes the compact interfacial contact between BOB and PML-Cu, which enables efficient electron transfer. After the contact, the electrons in conduction band (CB) of BOB spontaneously diffuse into PML-Cu and form electron depletion and accumulation layers on two sides of the interface. Influenced by the built-in electric field between the interfaces, a structure similar to the mutant inverse heterojunction is formed. Under light irradiation, the high concentration of electrons in CB of the host material BOB migrates toward PML-Cu and the potential barriers present in the interface can inhibit the reverse electron migration, resulting in the enrichment of photogenerated electrons in PML-Cu [47]. Usually, Br and O atoms have higher contribution to valence band (VB) of BOB. The halogen layer is relatively spatially independent from PML-Cu, leading most of the holes still remain in the VB of BOB. As a result, PBOB can provide more photogenerated electrons for CO_2_ reduction.

### CO_2_ Photoreduction Performance Evaluation

**Fig. 4:**
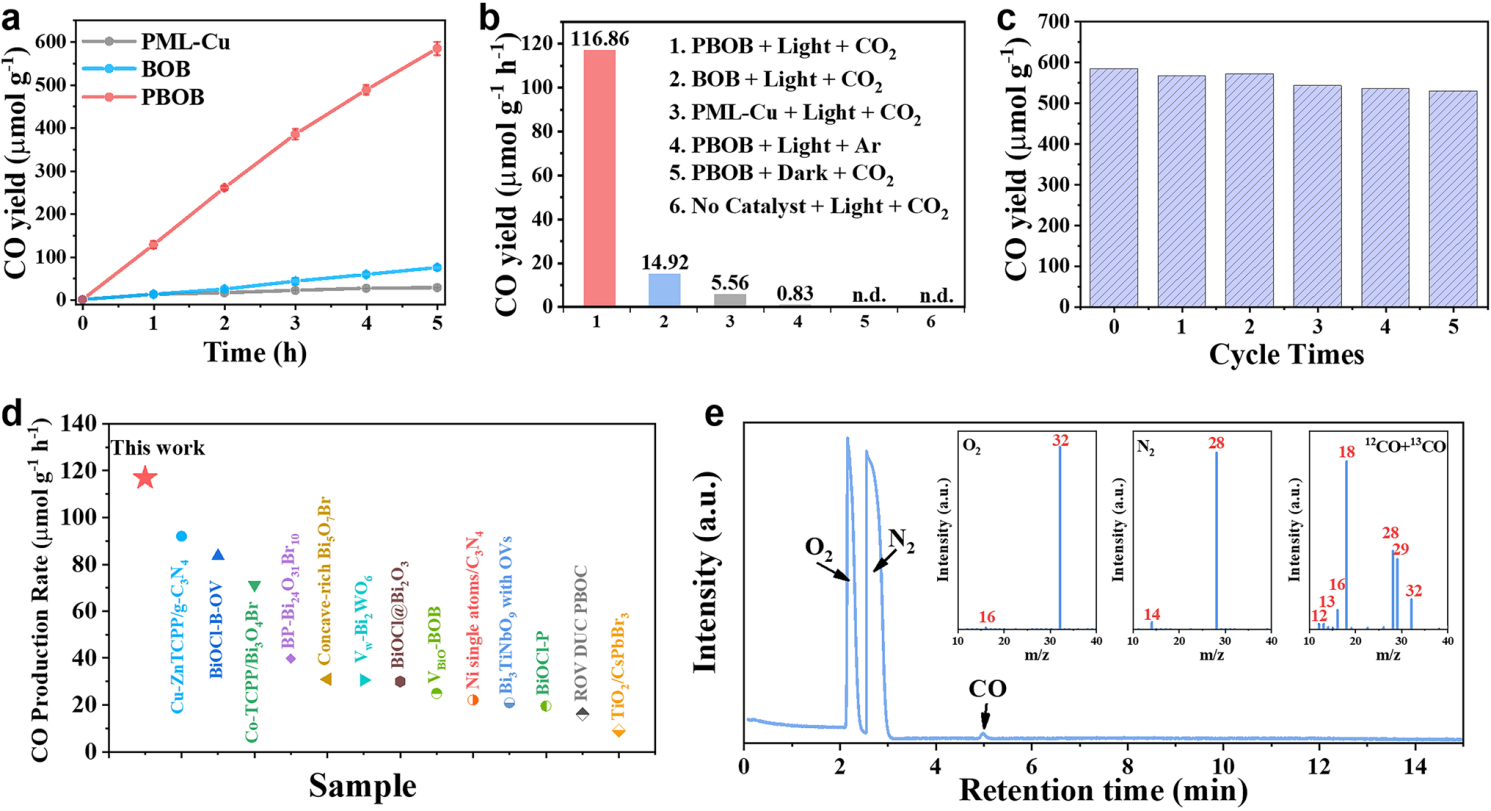
**a** Time courses of photocatalytic CO evolutions; **b** CO yield of the materials at different conditions; **c** Cycling stability test for PBOB; **d** Comparison of CO_2_ photoreduction evolution with other advanced materials; **e** Total ion chromatography in GC–MS measurement in CO_2_ photoreduction with ^12^CO_2_ and ^13^CO_2_ over PBOB, the inside is the corresponding mass spectra

The CO_2_ photoreduction performance of the photocatalyst was evaluated under pure water conditions without any sacrificial agent or co-catalyst (Labsolar-6A, Beijing Perfectlight). As shown in Fig. 4a, CO is the only C-containing product detected, and the CO_2_ photoreduction to CO of PBOB material is 584.3 μmol g^−1^ after 5 h, which is 7.83 times higher than that of BOB (74.6 μmol g^−1^) and 21.01 times of PML-Cu (27.8 μmol g^−1^). An experimental condition (Fig. 4b) was utilized to investigate the factors influencing CO_2_ photoreduction. Under Ar atmosphere, the CO yield of PBOB is 0.83 μmol g^−1^ h^−1^, which could be attributed to the conversion of CO_2_ adsorbed on the surface. Moreover, no CO production is observed in the system under conditions with either no light or no catalyst. These findings confirm the efficiency of the photocatalyst and establish that CO_2_ as a feedstock and light as an energy source are essential conditions for the reaction. Besides, the photocatalytic performance of mechanically mixed samples was also examined (Fig. S14). The CO yield of PML-Cu/BOB is only 62.57 μmol g^−1^ after 5 h, which is smaller than that of BOB and PBOB, verifying the necessity of bonding interface for surface interface dual polarization. After cycle tests for 5 times, the catalytic activity of PBOB can still maintain 90.60% (Fig. 4c), indicating PBOB can provide stable reduction capacity under the surface interface dual polarization strategy. Meanwhile, PBOB remains competitive compared to other advanced catalysts (Fig. 4d) [28, 35, 40, 48–57]. The photocatalytic half-reaction was also examined and a non-linear growth of O_2_ was detected in the TCD detector of the GC after a long reaction time, which may have produced other oxidation products. The solution after 5 h of reaction was further analyzed (Fig. S15), in which the generation of photocatalytic oxidation end products was detected [58]. Among them, the H_2_O_2_ production was 63.80, 4.26 and 0.10 μmol g^−1^ for PBOB, BOB and PML-Cu, respectively. The carbon source of the CO was analyzed by ^13^C isotope tracing experiments (Fig. 4e). The reaction was carried out using ^13^CO_2_ with ^12^CO_2_ as raw gas and GC–MS for reaction products. In the total ion flow diagram, the peaks at 2.1 and 2.5 min represent O_2_ (m/z = 32) and N_2_ (m/z = 28), respectively. The signals at m/z = 28 and 29 in the mass spectra corresponding to the peaks present at 4.94 min in the total ion flow diagram, which confirmed the production of ^12^CO and ^13^CO, indicating that CO_2_ can be effectively converted to CO through the photoreduction of PBOB.

### ***CO***_***2***_*** Photoreduction Mechanism Analysis***

**Fig. 5:**
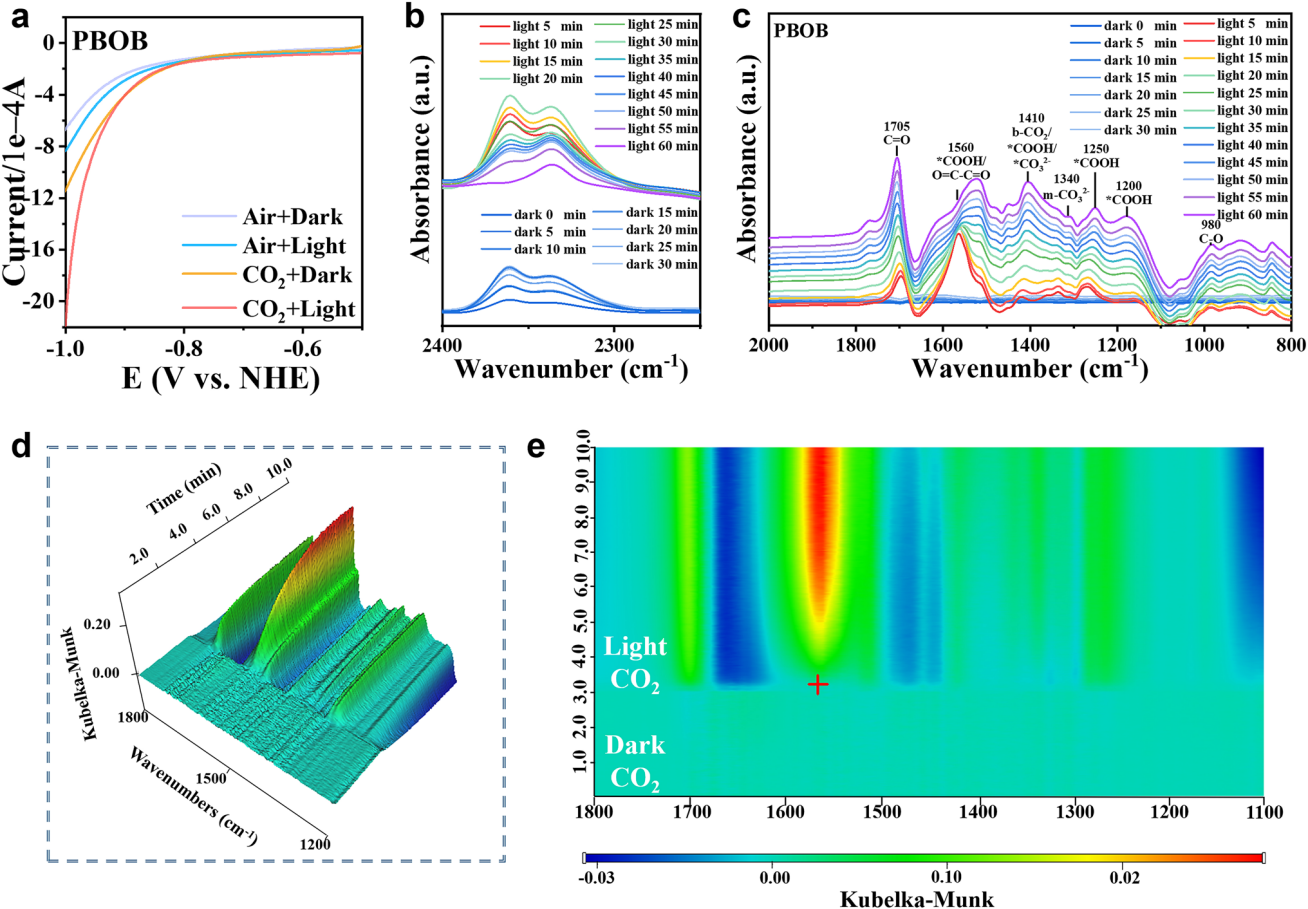
**a** LSV curves of PBOB under Ar and CO_2_; **b**, **c** In situ FTIR spectra for the CO_2_ reduction process; **d** 3D Time-resolved in situ MCT-SEIRAS FTIR spectra and **e** 2D contour color map of PBOB

The reactivity of the catalysts and the adsorption-activation-mass transfer process of CO_2_ molecules over the catalytic site are important factors affecting CO_2_ photoreduction. Linear sweep voltammetry (LSV) tests were respectively performed in Ar and CO_2_ gases to evaluate the priority of CO_2_ reduction and competitive hydrogen reaction (Figs. 5a and S16) [59]. The cathodic current generated under Ar atmosphere mainly originated from the hydrogen evolution reaction. Compared with BOB, PBOB has larger cathodic current. When CO_2_ was drummed into the reaction solution, the current density of PBOB was significantly enhanced, verifying that PBOB favor to perform the CO_2_ reduction reaction. During illumination, the further enhanced current intensity of PBOB indicates that light can effectively drive the reaction to proceed.

The CO_2_ photoreduction over PBOB surface was monitored by in situ Fourier transform infrared spectroscopy (Fig. 5b, c). The IR absorption peaks located at 2320 to 2370 cm^−1^ (asymmetric stretching of CO_2_) are significantly enhanced after the CO_2_ injection under dark, confirming the effective adsorption of CO_2_ on PBOB surface. The intensity of this double peak gradually decreases under continuous irradiation, indicating that CO_2_ is rapidly reduced as raw gas. During the photoreaction, the peak at 1340 cm^−1^ represents m-CO_3_^2−^, the fast-growing multiple IR peaks appearing at 1200, 1250, and 1560 cm^−1^ correspond to *COOH, which is the key intermediate in photocatalytic CO_2_ reduction [60–62]. Meanwhile, the characteristic peak for C = O at 1705 cm^−1^ is associated with the product CO. At the early stage of the photoreaction, the peak at 1705 cm^−1^ is significantly lower than that at 1560 cm^−1^. Then, the ratio of the two peaks gradually approach 1:1 and further increases after 30 min, indicating that the PBOB with rich surface-active Cu sites contribute to the rapid generation of *COOH and its gradual conversion into CO [48, 63]. Afterward, the catalyst surface was continuously scanned with time-resolved in situ MCT-SEIRAS FTIR (Fig. 5d, e). After passing CO_2_ in darkness, the appearance of a continuously increasing signal in the range of 1300–1660 cm^−1^ demonstrates the establishment of effective CO_2_ chemisorption on PBOB surface. During illumination, the formation of dark blue region indicates the rapid depletion of adsorbed CO_2_, while the continuous surge of red signal shows that light triggeres the efficient reduction of CO_2_ to CO, and thus verifies the rapid adsorption-diffusion-mass transfer process of CO_2_ on the PBOB surface. Based on the above results, the possible path of CO_2_ photoreduction on PBOB is proposed in Fig. S17.

**Fig. 6:**
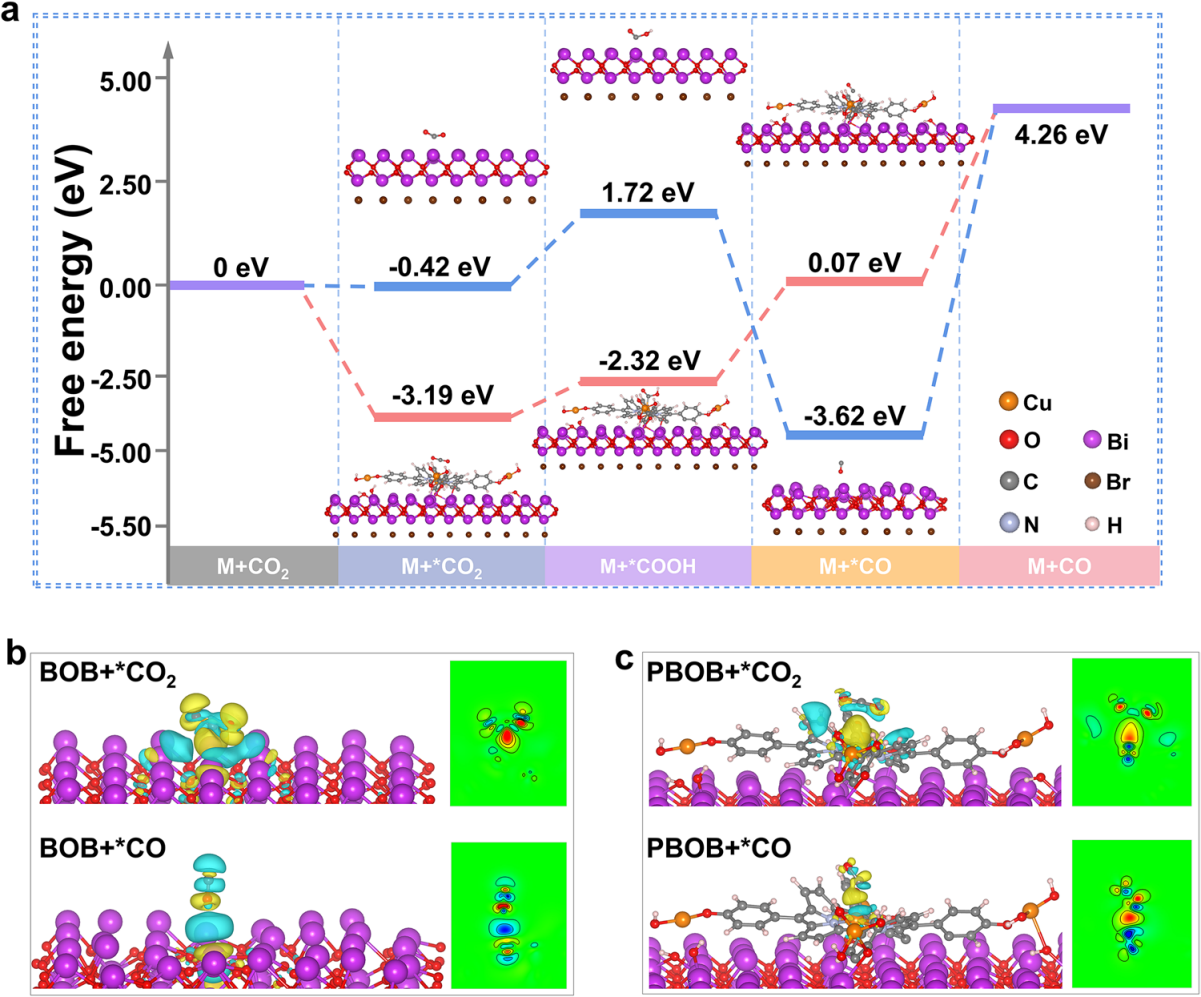
**a** Calculated Gibbs free-energy diagram for CO_2_ reduction to CO over BOB and PBOB; Electron density differences of CO_2_ and CO over **b** BOB and **c** PBOB

Gibbs free energy calculations were employed to obtain in-depth mechanistic understanding of this process. As shown in Fig. 6a, models for CO_2_ adsorption, adsorbed state intermediate COOH and CO desorption were constructed to investigate the ease with the typical process reaction occurring during CO_2_ reduction. Although the formation of both CO_2_ and CO in the adsorbed state on the BOB is spontaneous, it is very difficult to realize CO desorption, which needs up to 7.88 eV in energy. CO that fails to desorb in time will continue to occupy the active sites and inhibit the reaction. In addition to achieving the high density of active sites, the construction of PBOBs via the surface interface dual polarization strategy significantly reduces the activation energy barriers required for most of the reaction steps. The introduced PML-Cu greatly facilitate CO_2_ adsorption (from − 0.42 to − 3.19 eV) and reduce the activation energies for *COOH formation and the key steps CO desorption by 1.27 and 3.69 eV, respectively. As a result, due to the small difference of activation energy barrier between each individual reaction steps, the CO_2_ reduction for PBOB can proceed more smoothly. The electron transfer of CO_2_ and CO over the surface of the material during the critical step was probed by charge density difference (Fig. 6b, c), respectively. For the CO_2_ adsorption model, the electrons transferred to CO_2_ from Cu sites in PBOB are mainly enriched in C atoms. However, the electrons transferred from Bi atoms to C atoms in BOB are further dispersed toward O atoms via C=O bonds. This intramolecular charge transfer leads to a stronger bond energy of the C=O bond and a higher potential barrier required for bond breakage, which is not favorable for the conversion to *COOH. In the CO desorption model, O atom in CO molecule shows stronger affinity to the Bi site on BOB surface, while CO interact with PBOB through Cu and C atom. Due to the weaker electronegativity of C than O, the energy required for CO dissociation on PBOB surface is lower.

## Conclusions

Strain-induced surface interface dual polarization is developed to address the difficulties of insufficient active sites and slow interfacial charge transfer in photocatalysts. PML-Cu was constructed using Cu-TCPP as a substrate, and the PBOB catalyst was constructed by assembling PML-Cu with BOB through a strain engineering strategy. The Bi-O bonding interface formed built-in electric field between BOB and PML-Cu, triggering the electron transfer from CB of BOB to CB of PML-Cu and suppresses its reverse migration. The surface polarization of PML-Cu further promotes the electron converge in Cu atoms. The introduction of PML-Cu endows a high density of dispersed Cu active sites on the surface of PBOB, significantly promoting the adsorption and activation of CO_2_ and CO desorption. The resulting photocatalytic CO_2_ reduction to CO production rate of PBOB for 5 h reaches 584.3 μmol g^−1^, which is 7.83 times higher than that of BOB and 20.01 times of PML-Cu respectively. This work demonstrates the great potential of multistep polarization and active sites concentration construction for hybrid materials.

## Supplementary Information

Below is the link to the electronic supplementary material.

Supplementary file1 (PDF 698 kb)
