# Supplementary material for: Strain-Induced Surface Interface Dual Polarization Constructs PML-Cu/Bi12O17Br2 High-Density Active Sites for CO2 Photoreduction
Source: Nanomicro Lett. 2024 Jan 16;16:90. doi: 10.1007/s40820-023-01309-w (PMC10792156; doi:10.1007/s40820-023-01309-w)
Supplement: Supplementary file 1 — Supplementary file1 (PDF 698 kb) [file 40820_2023_1309_MOESM1_ESM.pdf]

Supporting Information for

Strain-Induced Surface Interface Dual Polarization Constructs PML-Cu/$Bi_{12}O_{17}Br_2$ High-Density Active Sites for CO_2 Photoreduction

Yi Zhang[1,4,#], Fangyu Guo[3,#], Jun Di[5,6,*], Keke Wang[2], Molly Meng-Jung Li[4], Jiayu Dai[3,*], Yuanbin She[2,*], Jiexiang Xia[1,*], Huaming Li[1]

[1] School of Chemistry and Chemical Engineering, Institute for Energy Research, School of the Environment and Safety Engineering, Jiangsu University, Zhenjiang 212013, P. R. China

[2] State Key Laboratory Breeding Base of Green Chemistry-Synthesis Technology, College of Chemical Engineering, Zhejiang University of Technology, Hangzhou 310014, P. R. China

[3] College of Science, National University of Defense Technology, Changsha 410073, P. R. China

[4] Department of Applied Physics, The Hong Kong Polytechnic University, Hung Hom, Hong Kong, P. R. China

[5] School of Chemistry and Chemical Engineering, National Special Superfine Powder Engineering Research Center, Nanjing University of Science and Technology, Nanjing 210094, P. R. China

[6] Key Laboratory of the Ministry of Education for Advanced Catalysis Materials, Zhejiang Normal University, Jinhua 321004, P. R. China

[#] Yi Zhang and Fangyu Guo contributed equally to this work.

*Corresponding authors. E-mail: xjx@ujs.edu.cn (J.X. Xia); dijun@njust.edu.cn (J. Di); jydai@nudt.edu.cn (J.Y. Dai); sheyb@zjut.edu.cn (Y.B. She)

S1 Experimental Section

S1.1 Synthesis of 5,10,15,20-Tetrakis(4-methoxycarbonylphenyl) Porphyrin

In a three-necked round-bottomed flask, 84.1 mmol of methyl 4-formylbenzoate was dissolved in 300.0 mL of propionic acid, the temperature was raised to 140 °C with stirring under N_2 and refluxed for 10.0 min, then 89.4 mmol of redistilled pyrrole was added dropwise within 5.0 min and the reaction continued to be refluxed under stirring for 2 h. The reaction was cooled down to room temperature, and the precipitate was collected by filtration and dissolved by adding dichloromethane. Subsequently, 500.0 mL of methanol was added and the solution was stirred for 1.0 h. The precipitated solid was filtered and washed with methanol, and then purified by silica gel column chromatography using silica gel (200~300 mesh) and eluent (V cyclohexane: V

dichloromethane = 4:1). Purple crystals can be obtained by desolvation under reduced pressure and vacuum drying.

S1.2 Synthesis of 5, 10, 15, 20-tetrakis(4-methoxycarbonylphenyl) Porphyrin Cobalt(II)

4 mmol of the above solid was taken and dissolved with 40 mmol of anhydrous cobalt acetate in 600 mL of DMF. Subsequently, the solution was stirred under N_2 protection for 10 min, heated to 150 °C and kept at reflux for 2 h. After cooling to room temperature, the solution was desolvated under reduced pressure to obtain the solid and dissolved in 100 mL of dichloromethane. The sample was washed several times with distilled water and the lower organic phase was extracted and dried with anhydrous sodium sulfate for 30 min. Subsequently, it was desolvated under reduced pressure, dissolved and chromatographed with 20 mL of dichloromethane (silica gel was used as stationary phase and dichloromethane as eluent). The solution was desolvated under reduced pressure and dried under vacuum at 70 °C for 5 h to obtain solid powder.

S1.3 Synthesis of Cobalt(II) 5, 10, 15, 20-tetrakis(4-carboxyphenyl) Porphyrin

In a round-bottomed flask, 0.8303 mmol of the above solid was taken and 46.95 mmol of KOH, 25 mL of THF, 25 mL of MeOH, 25 mL of H_2O were added sequentially, heated up to 85 °C and refluxed with stirring for 12 h. The solution was cooled to room temperature and depressurized. The sample was fully dissolved in distilled water and the pH was adjusted to between 2-3. Filtered under reduced pressure and washed several times, dried under vacuum at 70 °C for 5 h to obtain the solid product and labeled as Co-TCPP.

S1.4 Synthesis of Mechanically Mixed Sample (PML/BOB)

In an agate mortar, 60 mg of Bi_3O_4Br was combined with PML-Cu at a mass fraction of 0.05%. The mixture was then ground to produce a mechanically mixed sample of Bi_3O_4Br and PML-Cu, denoted as PML-Cu/BOB.

S2 Characterizations

The crystal structure information of the sample was obtained by XRD (*Shimadzu XRD-6000 diffractometer*) with Cu Kα radiation at room temperature. Raman Spectrometer (*American ThermoFisher DXR*) was applied to analyze the molecular structure. The structure analysis and identification of samples were determined by Infrared absorption spectrum and reflection spectrum (*NICOLET iS50 FT-IR*). The topography and size structure were presented through transmission electron microscopy (*TEM, JEOL JEM-2100F*). High power transmission data and element distribution information of crystals were presented through aberration-corrected high-angular annular dark-field scanning transmission electron microscope (HAADF-STEM) JEM-ARM200F. The light absorption characteristics of materials were recorded on a UV-vis spectrophotometer (*Shimadzu UV-2450*) in the diffuse reflectance pattern. X-ray photoelectron spectroscopy (XPS) analysis was measured on the *Shimadzu kratos AXIS UltraDLD*

system using the contaminant carbon (C1s= 284.6 eV) as a reference. All electrochemical tests were performed on a CHI 760B electrochemical system (Chenhua Instruments) in conventional three-electrode cell with Pt as the counter electrode, and Ag/AgCl/sat. KCl electrode as the reference electrode. The migration of photogenerated charges was analyzed through steady-state and transient fluorescence (*Edinburghinstruments, FS5*), the excitation and emission wavelength are 360 and 460 nm, respectively. In the laser-induced fluorescence experiment, a 360 nm laser light source was used to excite the sample placed in a quartz clamp, and the 475 nm broadband filter was equipped in front of the detector. The isotope-labeled experiment was conducted using $^{13}CO_2$ instead of $^{12}CO_2$, and the products were analyzed through gas chromatography-mass spectrometry (7890A and 5975C, Agilent). CO_2 TPD measurements were carried out on quantachrome autosorb-iQ-C chemisorption analyzer with a thermal conductivity detector.

S3 Methods

S3.1 CO_2 Photoreduction

The evaluation for the CO_2 photoreduction performance of the samples was conducted in a 500 mL all-glass automatic on-line trace gas analysis system (Labsolar-6A, Beijing Perfectlight). 30 mg of samples were added to 50 mL of water and then dispersed. The system was initially in a high-vacuum state, followed by the introduction of high-purity CO_2 until the pressure of 0.08 MPa. The experiments were carried out at 5 °C, utilizing a circulating water system to prevent thermal catalytic effects. A Xenon lamp source (PLS-SXE300D, Beijing Perfectlight, Lamp Power: 300 W) was used to provide the light source. The light source was positioned close to the light window and 10 cm away from the bottom, delivering a light intensity of 280 mW/cm^2 on the samples. The sampling interval was set at 1 h.

S3.2 Photocatalytic H_2O_2 Generation

The concentration of the hydrogen peroxide was measured based on ($3I^- + H_2O_2 + 2H^+ = I_3^- + 2H_2O$). Firstly, 0.1 M KI solution and 0.01 M $H_{24}Mo_7N_6O_{24}\cdot4H_2O$ were prepared respectively. A series of reference solutions with suitable concentrations of $N_2O_2\cdot H_2O$ were made to measure their UV-vis absorbance at 352 nm to obtain the calibration curve. Then, the reaction solution was collected in the tubes and centrifuged to precipitate the photocatalysts. And 500 μl of supernatant liquid was mixed with 2 ml of 0.1 M KI and 50 μl of 0.01 M $H_{24}Mo_7N_6O_{24}\cdot4H_2O$. After 10 min, the concentration of H_2O_2 in the reaction solution was calculated via the as-obtained calibration curve.

S3.3 In-situ Fourier Transform Infrared Measurement

All infrared spectra were recorded on a Nicolet iS50 FT-IR spectrometer (Thermo Electron) equipped with a liquid nitrogen cooled HgCdTe (MCT) detector. In-situ Fourier transform infrared (FTIR) studies were performed in the Praving Mantis DRIFTS accessory and reaction chamber. The reaction cell was equipped with a heater and a temperature controller, and a sample cup was installed in the center. The dome of

the DRIFTS battery had two quartz windows, and one ZnSe window allowed radiation transmission through fiber optics by connecting to a high uniformity integrated Xenon light source (PLS-FX300HU, Beijing Perfectlight). Before adsorption/desorption, the sample was purged with nitrogen (N_2) at 150 °C for 1 h to clean the catalyst surface. The temperature was then lowered to room temperature, and the background spectrum was collected in the presence of the sample. The sample was purged with CO_2 and steam for 30 min to reach adsorption equilibrium. Next, the FTIR spectra were obtained as a function of time to study the desorption/conversion kinetics of the reactants under visible light irradiation.

S3.4 Calculation Details

All density functional theory (DFT) calculations are implemented using the Vienna Ab Initio Simulation Package (VASP). The exchange-functional is applied utilizing the generalized gradient approximation (GGA) of Perdew-Burke-Ernzerhof (PBE) functional, and use the plane-wave basis with a kinetic energy cutoff of 500 eV to account for valence electrons. In order to better simulate the actual surface situation, the vacuum spacing in a direction perpendicular to the plane of the catalyst is 20 Å in order to separate the surface slab from its periodic duplicates. The BOB (001) surface models is a periodic structure of 2×2 (clean surface) and 3×3 (with PLM-Cu surface), respectively. Due to the large size of the computational model (more than three hundred atoms), the gamma point in Brillouin zone is performed for k-point sampling. The self-consistent calculations applied a convergence energy threshold of 10-5 eV. The equilibrium lattice constants were optimized with maximum stress on each atom within 0.02 eV/Å. The lower two layers of atoms are fixed, and the others allow relaxation.

S4 Supplementary Figures and Table

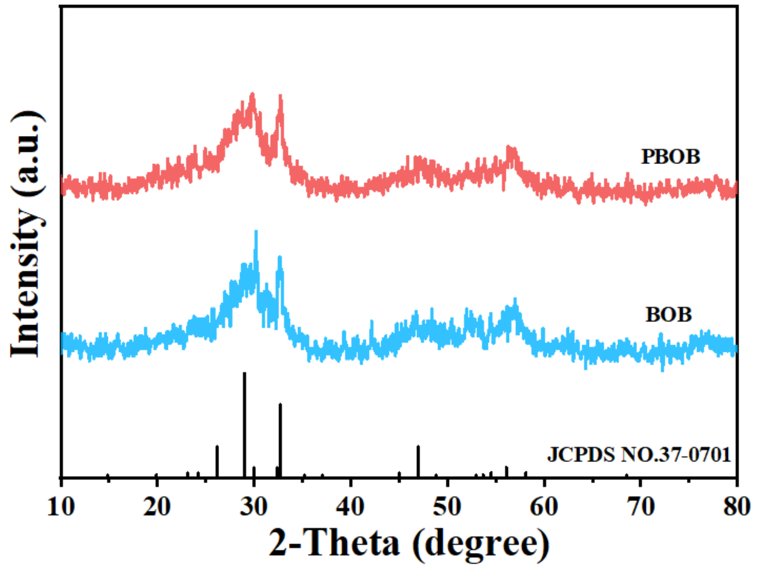

Fig. S1 XRD patterns of the materials

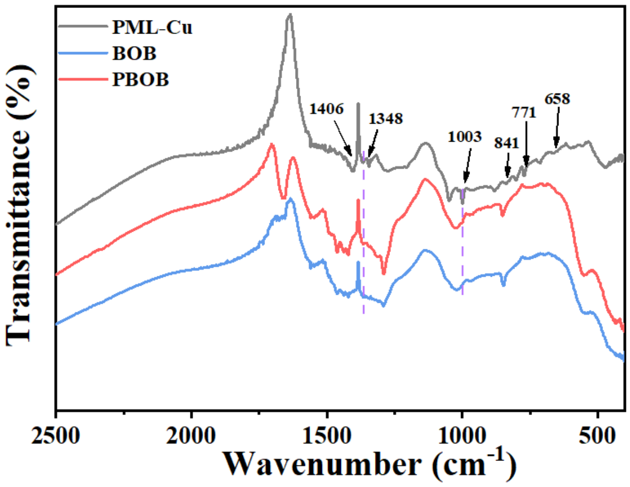

Fig. S2 FTIR spectrum of the materials

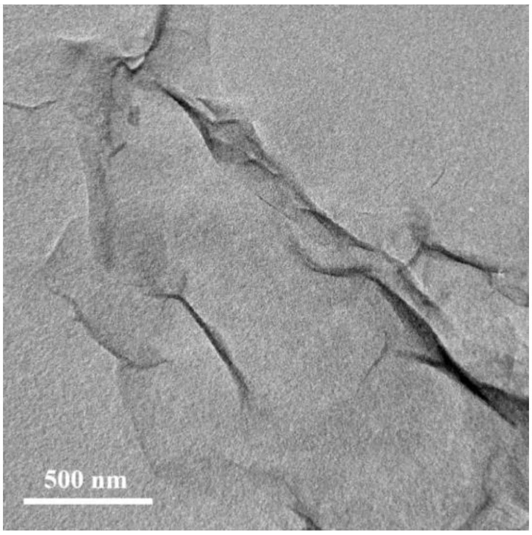

Fig. S3 TEM of PML-Cu

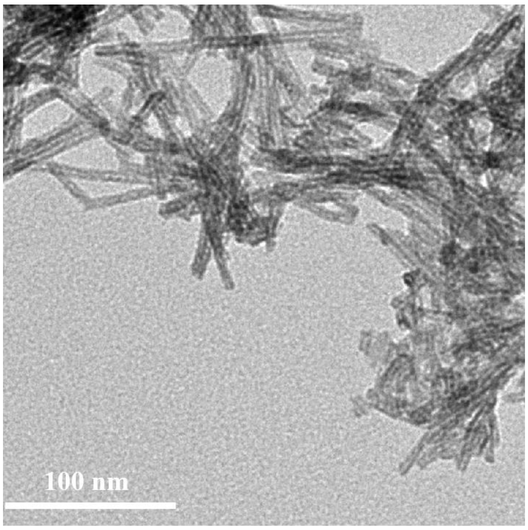

Fig. S4 TEM of BOB

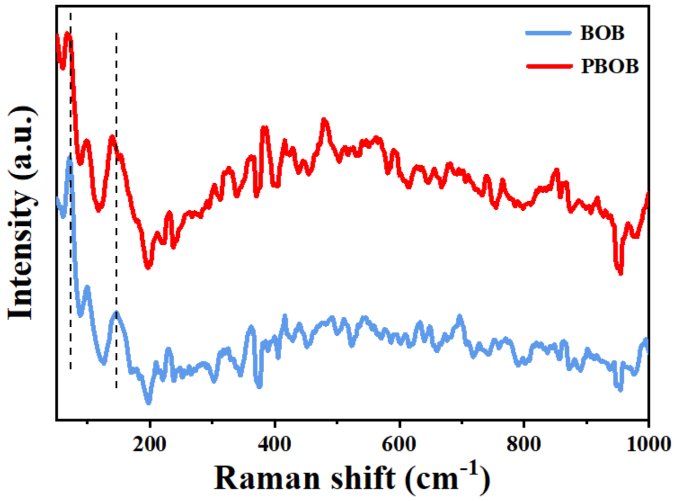

Fig. S5 Raman spectra of the materials

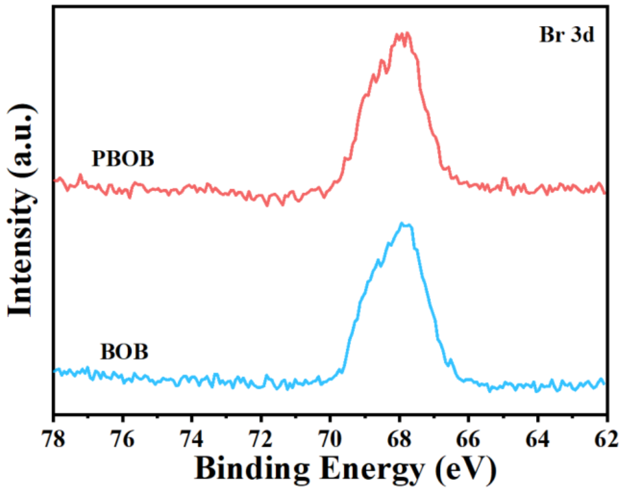

Fig. S6 XPS Br 3d spectra of the materials

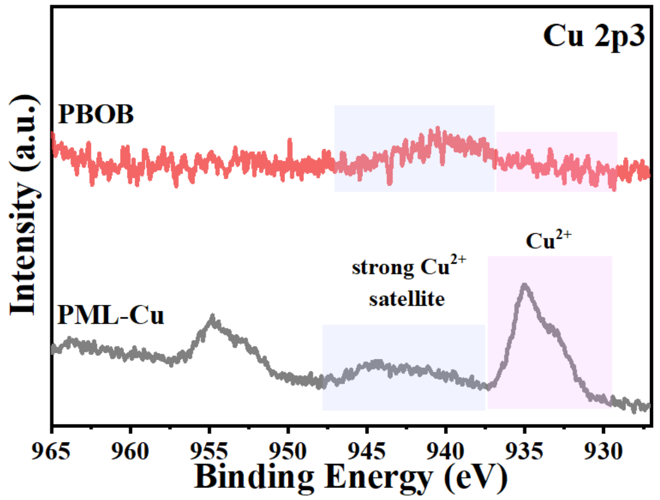

Fig. S7 XPS O 2p spectra of the materials

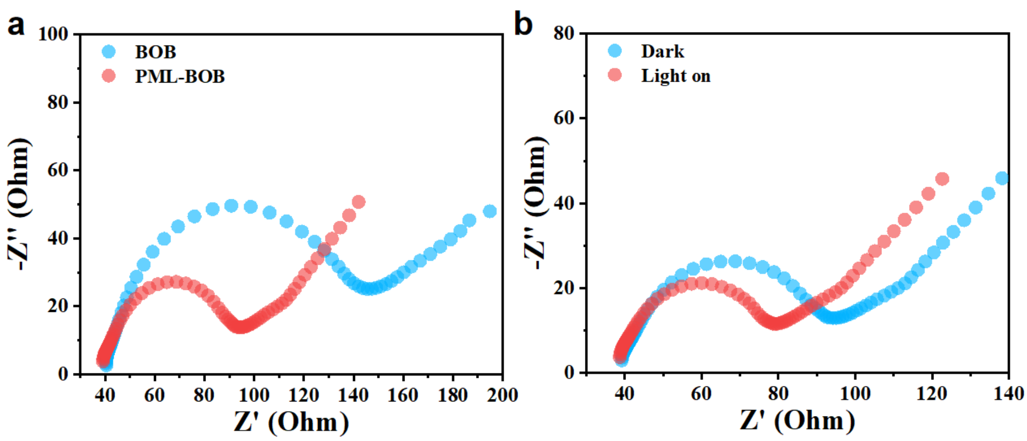

Fig. S8 EIS spectra of **a** BOB and PBOB; **b** PBOB under dark and light

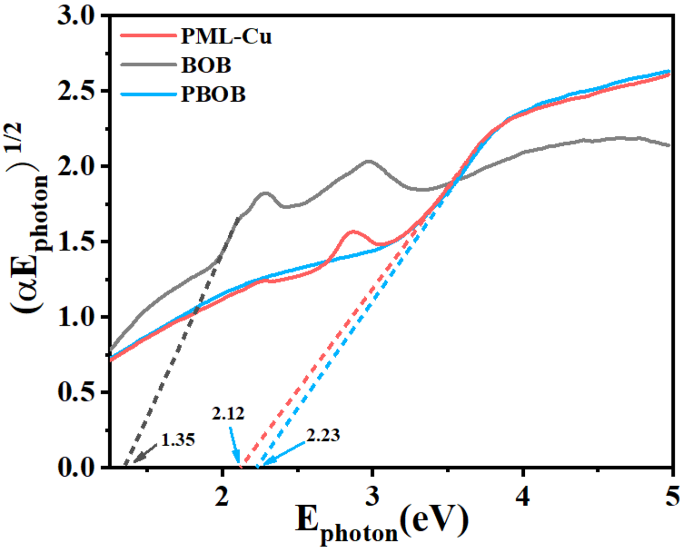

Fig. S9 Tauc plots of the materials

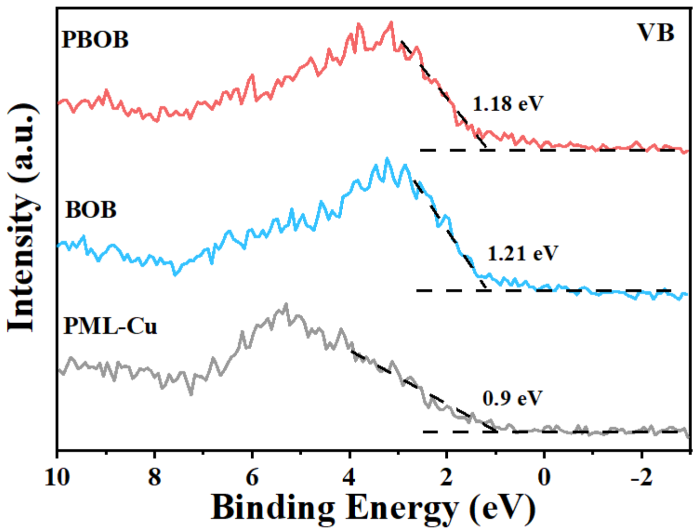

Fig. S10 VB XPS spectra of the materials

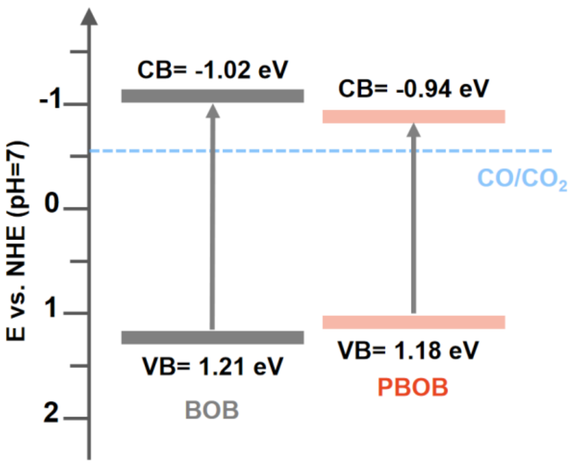

Fig. S11 Band structure of BOB and PBOB

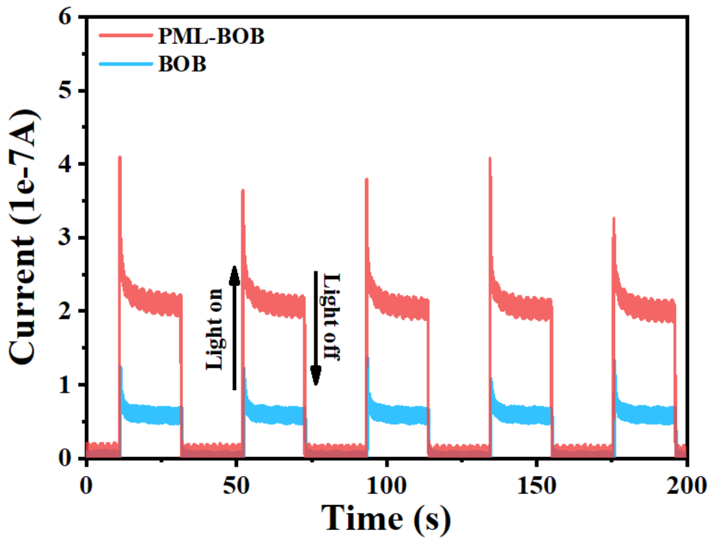

Fig. S12 Transient photocurrents of the materials

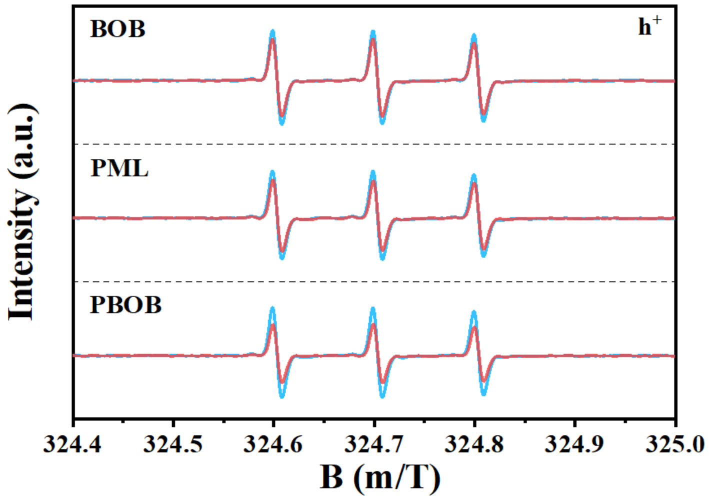

Fig. S13 ESR spectra for h^+ of the materials

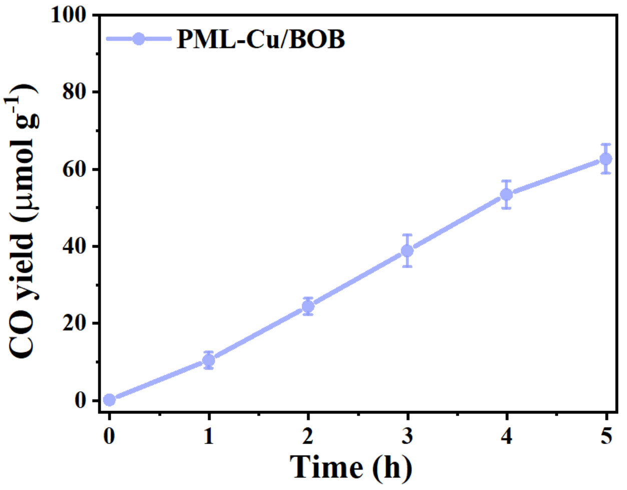

Fig. S14 Time courses of photocatalytic CO evolution of PML-Cu/BOB

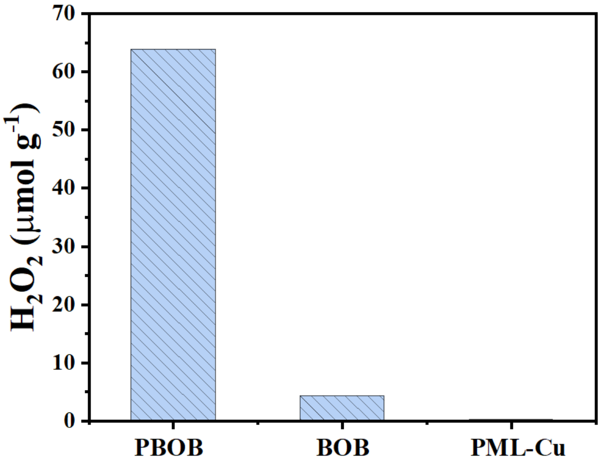

Fig. S15 H_2O_2 concentration of the materials

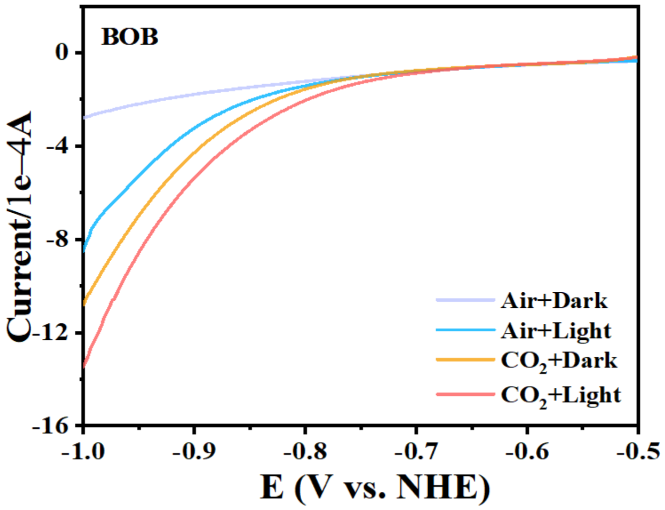

Fig. S16 LSV curves of BOB under Ar and CO_2

$$* + CO_2(g) + H^+ + e^- \rightarrow *COOH \qquad (1)$$

$$*COOH + H^+ + e^- \rightarrow *CO + H_2O \qquad (2)$$

$$*CO \rightarrow CO(g)\uparrow + * \qquad (3)$$

Fig. S17 Possible reaction path for CO_2 photoreduction (the asterisks denote catalytically active sites)

Table S1 Transient fluorescence fitting curve table

Sample	τ_1/ns (rel%)	τ_2/ns (rel%)	τ_3/ns (rel%)	τ_s/ns
**BOB**	0.9520 (67.90)	0.9521 (28.38)	11.4525 (3.72)	1.3426
**PBOB**	0.9882 (53.90)	0.9883 (41.08)	12.0261 (5.02)	1.5423
